# 6-Meth­oxy-2,3,4,9-tetra­hydro-1*H*-carbazol-1-one

**DOI:** 10.1107/S1600536808007885

**Published:** 2008-03-29

**Authors:** M. Sridharan, K. J. Rajendra Prasad, A. Thomas Gunaseelan, A. Thiruvalluvar, A. Linden

**Affiliations:** a Department of Chemistry, Bharathiar University, Coimbatore 641 046, Tamilnadu, India; b PG Research Department of Physics, Rajah Serfoji Government College (Autonomous), Thanjavur 613 005, Tamilnadu, India; c Institute of Organic Chemistry, University of Zurich, Winterthurerstrasse 190, CH-8057 Zürich, Switzerland

## Abstract

The carbazole unit of the title mol­ecule, C_13_H_13_NO_2_, is not planar. The dihedral angle between the benzene ring and the pyrrole ring is 1.69 (6)°. The cyclo­hexene ring adopts an envelope conformation. Inter­molecular C—H⋯O and N—H⋯O hydrogen bonds are present in the crystal structure. A C—H⋯π inter­action, involving the benzene ring, is also found in the crystal structure.

## Related literature

For related literature, see: Bhattacharya & Chakraborty (1987 ▶); Chakraborty & Roy (1991 ▶); Chakraborty (1993 ▶); Knolker (1986 ▶); Lescot *et al.* (1986 ▶); Hook *et al.* (1990 ▶); Hirata *et al.* (1999 ▶); Kapil (1971 ▶); Knolker & Reddy (2002 ▶); Sowmithran & Rajendra Prasad (1986 ▶); Rajendra Prasad & Vijayalakshmi (1994 ▶). Gunaseelan *et al.* (2007*a* ▶,*b* ▶) and Thiruvalluvar *et al.* (2007 ▶) have reported the crystal structures of substituted carbazole derivatives, in which the carbazole units are not planar.
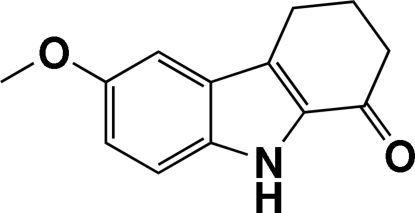


## Experimental

### 

#### Crystal data

C_13_H_13_NO_2_
*M* *_r_* = 215.24
Monoclinic, 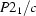

*a* = 9.0627 (2) Å
*b* = 14.0285 (3) Å
*c* = 8.5506 (2) Å
β = 101.815 (1)°
*V* = 1064.06 (4) Å^3^
*Z* = 4
Mo *K*α radiation
μ = 0.09 mm^−1^
*T* = 160 (1) K
0.35 × 0.28 × 0.13 mm

#### Data collection

Nonius KappaCCD area-detector diffractometer
Absorption correction: none
28554 measured reflections
3077 independent reflections
2601 reflections with *I* > 2σ(*I*)
*R*_int_ = 0.038

#### Refinement

*R*[*F* ^2^ > 2σ(*F* ^2^)] = 0.043
*wR*(*F* ^2^) = 0.145
*S* = 1.12
3077 reflections
149 parameters
H atoms treated by a mixture of independent and constrained refinement
Δρ_max_ = 0.33 e Å^−3^
Δρ_min_ = −0.24 e Å^−3^

### 

Data collection: *COLLECT* (Nonius, 2000 ▶); cell refinement: *DENZO-SMN* (Otwinowski & Minor, 1997 ▶); data reduction: *DENZO-SMN* and *SCALEPACK* (Otwinowski & Minor, 1997 ▶); program(s) used to solve structure: *SHELXS97* (Sheldrick, 2008 ▶); program(s) used to refine structure: *SHELXL97* (Sheldrick, 2008 ▶); molecular graphics: *ORTEP-3* (Farrugia, 1997 ▶); software used to prepare material for publication: *PLATON* (Spek, 2003 ▶).

## Supplementary Material

Crystal structure: contains datablocks global, I. DOI: 10.1107/S1600536808007885/wn2245sup1.cif

Structure factors: contains datablocks I. DOI: 10.1107/S1600536808007885/wn2245Isup2.hkl

Additional supplementary materials:  crystallographic information; 3D view; checkCIF report

## Figures and Tables

**Table 1:** Hydrogen-bond geometry (Å, °)

*D*—H⋯*A*	*D*—H	H⋯*A*	*D*⋯*A*	*D*—H⋯*A*
N9—H9⋯O1^i^	0.948 (17)	1.918 (17)	2.8313 (14)	161.2 (15)
C2—H2*A*⋯O2^ii^	0.99	2.52	3.4962 (15)	169
C4—H4*B*⋯*Cg*^iii^	0.99	2.57	3.492 (1)	156

Symmetry codes: (i) 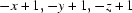; (ii) 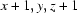; (iii) 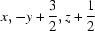. *Cg* is the centroid of the benzene ring.

**Fig. 1.**
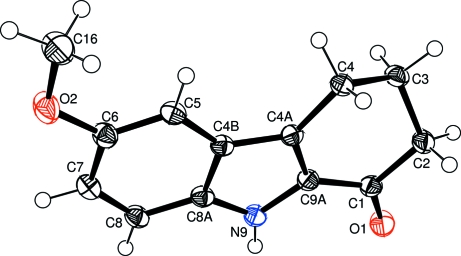
The molecular structure of the title compound, showing the atom-numbering scheme and displacement ellipsoids drawn at the 50% probability level. Hydrogen atoms are represented by spheres of arbitrary radius.

**Fig. 2.**
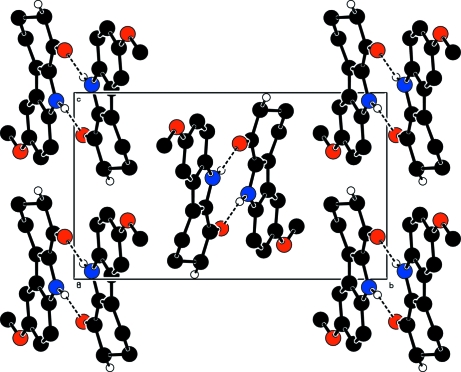
The molecular packing of the title compound, viewed down the a axis. Dashed lines indicate hydrogen bonds. H atoms not involved in hydrogen bonding have been omitted.

## supplementary crystallographic information

### Comment

Heterocylic compounds are encountered in a very large number of groups of organic compounds. They play a vital role in the metabolism of all living cells, which are widely distributed in nature and are essential to life. Among them the carbazole heterocycles have emerged as an important class, based on their fascinating structure and high degree of biological activities (Bhattacharya & Chakraborty,1987; Chakraborty & Roy, 1991; Chakraborty, 1993). A number of carbazole alkaloids with intriguing novel structures and useful biological activities were isolated from natural sources over the past decades; these attracted chemists to frame novel synthetic strategies towards the synthesis of carbazole and its derivatives (Knolker,1986; Lescot *et al.*, 1986). These alkaloids represent a new and interesting variant in the large number of indole alkaloids, which have yielded several important drugs. Several reports have appeared on the synthesis of carbazole derivatives, in connection with the search for newer physiologically active compounds (Hook *et al.*, 1990; Hirata *et al.*, 1999; Kapil, 1971; Knolker & Reddy, 2002). The preparation of 1-oxo compounds via their corresponding hydrazones have been reported (Sowmithran & Rajendra Prasad, 1986; Rajendra Prasad & Vijayalakshmi, 1994).

Gunaseelan *et al.* (2007*a*,b) and Thiruvalluvar *et al.* (2007) have reported the crystal structures of substituted carbazole derivatives, in which the carbazole units are not planar. The molecular structure of the title compound, with atomic numbering scheme, is shown in Fig. 1. The carbazole unit of the title molecule is not planar. The dihedral angle between the benzene ring and the pyrrole ring is 1.69 (6)°. The cyclohexene ring adopts an envelope conformation. Intermolecular C2—H2A···O2(*x* + 1, *y*, *z* + 1) and N9—H9···O1(-*x* + 1, -*y* + 1, -*z* + 1) hydrogen bonds are present in the crystal structure (Fig. 2). A C4—H4B···π(*x*, 3/2 - *y*,1/2 + *z*) interaction involving the benzene ring is also found in the structure, .

### Experimental

A solution of 2-(2-(4-methoxyphenyl)hydrazono)cyclohexanone (232 mg, 0.001 mol) in a mixture of acetic acid (20 ml) and hydrochloric acid (5 ml) was refluxed on an oil bath pre-heated to 398-403 K for 2 h. The reaction was monitored by TLC. After completion of the reaction the contents were cooled and poured on to cold water with stirring. The brown solid which separated was purified by passing through a column of silica gel and eluting with a (95:5) petroleum ether-ethyl acetate mixture, yielding the title compound (144 mg, 67%). The compound thus obtained was recrystallized using ethanol.

### Refinement

The H atom bonded to N9 was located in a difference Fourier map and refined isotropically. Other H atoms were positioned geometrically and allowed to ride on their parent atoms, with C—H = 0.95–0.99 Å and U_iso_(H) = xU_eq_(parent atom), where x = 1.5 for methyl and 1.2 for all other carbon-bound H atoms.

### Crystal data

**Table:**

C_13_H_13_NO_2_	*F*_000_ = 456
*M**_r_* = 215.24	*D*_x_ = 1.344 Mg m^−^^3^
Monoclinic, *P*2_1_/*c*	Melting point: 536 K
Hall symbol: -P 2ybc	Mo *K*α radiation λ = 0.71073 Å
*a* = 9.0627 (2) Å	Cell parameters from 3175 reflections
*b* = 14.0285 (3) Å	θ = 2.0–30.0º
*c* = 8.5506 (2) Å	µ = 0.09 mm^−^^1^
β = 101.815 (1)º	*T* = 160 (1) K
*V* = 1064.06 (4) Å^3^	Tablet, colourless
*Z* = 4	0.35 × 0.28 × 0.13 mm

### Data collection

**Table:**

Nonius KappaCCD area-detector diffractometer	3077 independent reflections
Radiation source: Nonius FR590 sealed tube generator	2601 reflections with *I* > 2σ(*I*)
Monochromator: horizontally mounted graphite crystal	*R*_int_ = 0.038
Detector resolution: 9 pixels mm^-1^	θ_max_ = 30.0º
*T* = 160(1) K	θ_min_ = 2.3º
φ and ω scans with κ offsets	*h* = −12→12
Absorption correction: none	*k* = 0→19
28554 measured reflections	*l* = 0→12

### Refinement

**Table:**

Refinement on *F*^2^	Secondary atom site location: difference Fourier map
Least-squares matrix: full	Hydrogen site location: inferred from neighbouring sites
*R*[*F*^2^ > 2σ(*F*^2^)] = 0.043	H atoms treated by a mixture of independent and constrained refinement
*wR*(*F*^2^) = 0.146	*w* = 1/[σ^2^(*F*_o_^2^) + (0.0825*P*)^2^ + 0.2332*P*] where *P* = (*F*_o_^2^ + 2*F*_c_^2^)/3
*S* = 1.12	(Δ/σ)_max_ < 0.001
3077 reflections	Δρ_max_ = 0.33 e Å^−^^3^
149 parameters	Δρ_min_ = −0.24 e Å^−^^3^
Primary atom site location: structure-invariant direct methods	Extinction correction: none

### Special details

**Table:**

Experimental. Solvent used: EtOH Cooling Device: Oxford Cryosystems Cryostream 700 Crystal mount: glued on a glass fibre Mosaicity (°.): 0.742 (2) Frames collected: 359 Seconds exposure per frame: 100 Degrees rotation per frame: 2.0 Crystal-Detector distance (mm): 30.0
Geometry. Bond distances, angles *etc*. have been calculated using the rounded fractional coordinates. All su's are estimated from the variances of the (full) variance-covariance matrix. The cell e.s.d.'s are taken into account in the estimation of distances, angles and torsion angles
Refinement. Refinement of *F*^2^ against ALL reflections. The weighted *R*-factor *wR* and goodness of fit *S* are based on *F*^2^, conventional *R*-factors *R* are based on *F*, with *F* set to zero for negative *F*^2^. The threshold expression of *F*^2^ > σ(*F*^2^) is used only for calculating *R*-factors(gt) *etc*. and is not relevant to the choice of reflections for refinement. *R*-factors based on *F*^2^ are statistically about twice as large as those based on *F*, and *R*- factors based on ALL data will be even larger.

### Fractional atomic coordinates and isotropic or equivalent isotropic displacement parameters (Å^2^)

**Table:**

	*x*	*y*	*z*	*U*_iso_*/*U*_eq_	
O1	0.53223 (10)	0.52978 (7)	0.72771 (11)	0.0324 (3)	
O2	−0.29638 (10)	0.67151 (8)	0.18475 (11)	0.0354 (3)	
N9	0.27900 (11)	0.55709 (7)	0.45710 (11)	0.0229 (3)	
C1	0.41200 (13)	0.56555 (8)	0.74513 (13)	0.0229 (3)	
C2	0.38660 (13)	0.59515 (9)	0.90781 (13)	0.0251 (3)	
C3	0.27324 (13)	0.67715 (8)	0.90149 (13)	0.0236 (3)	
C4	0.12214 (12)	0.65469 (8)	0.79020 (12)	0.0213 (3)	
C4A	0.14868 (12)	0.61976 (7)	0.63321 (12)	0.0196 (3)	
C4B	0.05307 (12)	0.61980 (7)	0.47874 (13)	0.0197 (3)	
C5	−0.09638 (12)	0.65107 (8)	0.42156 (13)	0.0219 (3)	
C6	−0.15394 (12)	0.64344 (8)	0.25953 (13)	0.0241 (3)	
C7	−0.06690 (13)	0.60611 (8)	0.15394 (13)	0.0253 (3)	
C8	0.07867 (13)	0.57456 (8)	0.20802 (13)	0.0236 (3)	
C8A	0.13856 (12)	0.58090 (7)	0.37241 (13)	0.0208 (3)	
C9A	0.28448 (12)	0.58046 (8)	0.61527 (13)	0.0213 (3)	
C16	−0.38951 (15)	0.71146 (13)	0.28342 (18)	0.0441 (5)	
H2A	0.48420	0.61484	0.97491	0.0301*	
H2B	0.34978	0.53939	0.95963	0.0301*	
H3A	0.25628	0.68946	1.01044	0.0283*	
H3B	0.31607	0.73572	0.86390	0.0283*	
H4A	0.06803	0.60539	0.83938	0.0256*	
H4B	0.05882	0.71278	0.77373	0.0256*	
H5	−0.15509	0.67641	0.49192	0.0262*	
H7	−0.10988	0.60272	0.04300	0.0303*	
H8	0.13633	0.54942	0.13648	0.0284*	
H9	0.3587 (19)	0.5296 (12)	0.416 (2)	0.038 (4)*	
H16A	−0.48742	0.72886	0.21742	0.0661*	
H16B	−0.34089	0.76845	0.33697	0.0661*	
H16C	−0.40428	0.66454	0.36374	0.0661*	

### Atomic displacement parameters (Å^2^)

**Table:**

	*U*^11^	*U*^22^	*U*^33^	*U*^12^	*U*^13^	*U*^23^
O1	0.0269 (5)	0.0436 (5)	0.0274 (4)	0.0137 (4)	0.0075 (3)	−0.0016 (4)
O2	0.0230 (4)	0.0513 (6)	0.0293 (5)	0.0061 (4)	−0.0008 (3)	−0.0103 (4)
N9	0.0239 (5)	0.0262 (5)	0.0202 (4)	0.0049 (3)	0.0084 (3)	−0.0009 (3)
C1	0.0245 (5)	0.0232 (5)	0.0222 (5)	0.0045 (4)	0.0076 (4)	0.0004 (4)
C2	0.0249 (5)	0.0302 (6)	0.0205 (5)	0.0067 (4)	0.0054 (4)	−0.0010 (4)
C3	0.0235 (5)	0.0252 (5)	0.0227 (5)	0.0028 (4)	0.0064 (4)	−0.0045 (4)
C4	0.0224 (5)	0.0233 (5)	0.0196 (5)	0.0030 (4)	0.0073 (4)	−0.0010 (4)
C4A	0.0215 (5)	0.0188 (5)	0.0199 (5)	0.0012 (3)	0.0073 (4)	0.0012 (3)
C4B	0.0215 (5)	0.0182 (5)	0.0205 (5)	−0.0004 (4)	0.0071 (4)	−0.0004 (3)
C5	0.0212 (5)	0.0223 (5)	0.0232 (5)	−0.0014 (4)	0.0071 (4)	−0.0022 (4)
C6	0.0208 (5)	0.0260 (5)	0.0249 (5)	−0.0018 (4)	0.0036 (4)	−0.0033 (4)
C7	0.0274 (6)	0.0272 (5)	0.0211 (5)	−0.0025 (4)	0.0045 (4)	−0.0034 (4)
C8	0.0276 (5)	0.0245 (5)	0.0204 (5)	−0.0009 (4)	0.0089 (4)	−0.0025 (4)
C8A	0.0231 (5)	0.0200 (5)	0.0210 (5)	0.0001 (4)	0.0085 (4)	−0.0005 (3)
C9A	0.0232 (5)	0.0222 (5)	0.0197 (5)	0.0030 (4)	0.0073 (4)	0.0002 (4)
C16	0.0261 (6)	0.0610 (10)	0.0421 (8)	0.0118 (6)	0.0000 (5)	−0.0185 (7)

### Geometric parameters (Å, °)

**Table:**

O1—C1	1.2360 (15)	C6—C7	1.4154 (16)
O2—C6	1.3756 (15)	C7—C8	1.3785 (17)
O2—C16	1.4247 (18)	C8—C8A	1.4021 (15)
N9—C8A	1.3706 (15)	C2—H2A	0.9900
N9—C9A	1.3826 (14)	C2—H2B	0.9900
N9—H9	0.948 (17)	C3—H3A	0.9900
C1—C9A	1.4446 (16)	C3—H3B	0.9900
C1—C2	1.5138 (16)	C4—H4A	0.9900
C2—C3	1.5359 (17)	C4—H4B	0.9900
C3—C4	1.5318 (16)	C5—H5	0.9500
C4—C4A	1.4940 (14)	C7—H7	0.9500
C4A—C4B	1.4236 (15)	C8—H8	0.9500
C4A—C9A	1.3854 (16)	C16—H16A	0.9800
C4B—C8A	1.4189 (15)	C16—H16B	0.9800
C4B—C5	1.4124 (16)	C16—H16C	0.9800
C5—C6	1.3810 (15)		
			
O1···N9	2.9314 (13)	C16···H3B^viii^	2.9800
O1···N9^i^	2.8313 (14)	H2A···O2^x^	2.5200
O1···H9	2.804 (17)	H2A···C16^x^	2.9800
O1···H2B^ii^	2.8400	H2A···H16A^x^	2.5900
O1···H9^i^	1.918 (17)	H2B···O1^ii^	2.8400
O2···H2A^iii^	2.5200	H2B···C2^ii^	3.0700
N9···O1	2.9314 (13)	H3A···C8^xi^	3.0300
N9···O1^i^	2.8313 (14)	H3A···H8^xi^	2.5900
C1···C16^iv^	3.590 (2)	H3B···C9A	3.0200
C2···C2^ii^	3.5370 (17)	H3B···C8A^v^	3.0400
C3···C8A^v^	3.5983 (15)	H3B···C16^iv^	2.9800
C4B···C4B^vi^	3.5353 (14)	H3B···H16A^iv^	2.4300
C6···C9A^vi^	3.5958 (16)	H4A···C7^vi^	2.9700
C8A···C3^vii^	3.5983 (15)	H4A···C8^vi^	2.8400
C9A···C6^vi^	3.5958 (16)	H4B···C4B^v^	2.9400
C16···C1^viii^	3.590 (2)	H4B···C5^v^	2.8200
C1···H16A^iv^	3.0500	H4B···C6^v^	2.7800
C1···H9^i^	3.028 (17)	H4B···C7^v^	2.8900
C2···H2B^ii^	3.0700	H4B···C8^v^	3.0500
C4B···H4B^vii^	2.9400	H4B···C8A^v^	3.0600
C5···H16C	2.7400	H5···C16	2.5300
C5···H4B^vii^	2.8200	H5···H16B	2.3100
C5···H16B	2.7400	H5···H16C	2.3100
C6···H4B^vii^	2.7800	H8···H3A^ix^	2.5900
C7···H4B^vii^	2.8900	H9···O1	2.804 (17)
C7···H4A^vi^	2.9700	H9···O1^i^	1.918 (17)
C8···H4B^vii^	3.0500	H9···C1^i^	3.028 (17)
C8···H4A^vi^	2.8400	H16A···H2A^iii^	2.5900
C8···H3A^ix^	3.0300	H16A···C1^viii^	3.0500
C8A···H4B^vii^	3.0600	H16A···H3B^viii^	2.4300
C8A···H3B^vii^	3.0400	H16B···C5	2.7400
C9A···H3B	3.0200	H16B···H5	2.3100
C16···H2A^iii^	2.9800	H16C···C5	2.7400
C16···H5	2.5300	H16C···H5	2.3100
			
C6—O2—C16	116.79 (10)	C1—C2—H2A	109.00
C8A—N9—C9A	107.61 (9)	C1—C2—H2B	109.00
C9A—N9—H9	125.6 (10)	C3—C2—H2A	109.00
C8A—N9—H9	126.8 (10)	C3—C2—H2B	109.00
O1—C1—C9A	123.53 (10)	H2A—C2—H2B	108.00
O1—C1—C2	121.72 (10)	C2—C3—H3A	109.00
C2—C1—C9A	114.73 (10)	C2—C3—H3B	109.00
C1—C2—C3	113.55 (9)	C4—C3—H3A	109.00
C2—C3—C4	111.98 (9)	C4—C3—H3B	109.00
C3—C4—C4A	109.74 (9)	H3A—C3—H3B	108.00
C4B—C4A—C9A	106.45 (9)	C3—C4—H4A	110.00
C4—C4A—C4B	130.85 (10)	C3—C4—H4B	110.00
C4—C4A—C9A	122.69 (10)	C4A—C4—H4A	110.00
C5—C4B—C8A	120.56 (10)	C4A—C4—H4B	110.00
C4A—C4B—C8A	106.61 (9)	H4A—C4—H4B	108.00
C4A—C4B—C5	132.82 (10)	C4B—C5—H5	121.00
C4B—C5—C6	117.50 (10)	C6—C5—H5	121.00
O2—C6—C5	124.74 (10)	C6—C7—H7	119.00
O2—C6—C7	113.70 (10)	C8—C7—H7	119.00
C5—C6—C7	121.55 (10)	C7—C8—H8	121.00
C6—C7—C8	121.66 (10)	C8A—C8—H8	121.00
C7—C8—C8A	117.60 (10)	O2—C16—H16A	109.00
N9—C8A—C8	129.88 (10)	O2—C16—H16B	109.00
C4B—C8A—C8	121.11 (10)	O2—C16—H16C	109.00
N9—C8A—C4B	108.97 (9)	H16A—C16—H16B	109.00
C1—C9A—C4A	124.16 (10)	H16A—C16—H16C	109.00
N9—C9A—C1	125.48 (10)	H16B—C16—H16C	109.00
N9—C9A—C4A	110.36 (10)		
			
C16—O2—C6—C5	0.21 (18)	C4B—C4A—C9A—N9	0.74 (12)
C16—O2—C6—C7	−178.89 (12)	C9A—C4A—C4B—C8A	−0.84 (11)
C9A—N9—C8A—C8	177.52 (11)	C4—C4A—C9A—N9	−178.17 (10)
C8A—N9—C9A—C4A	−0.35 (12)	C4—C4A—C4B—C8A	177.95 (10)
C9A—N9—C8A—C4B	−0.20 (12)	C9A—C4A—C4B—C5	−179.48 (11)
C8A—N9—C9A—C1	179.00 (10)	C5—C4B—C8A—N9	179.49 (10)
C9A—C1—C2—C3	−29.21 (14)	C4A—C4B—C8A—N9	0.65 (12)
O1—C1—C2—C3	152.17 (11)	C5—C4B—C8A—C8	1.54 (16)
C2—C1—C9A—N9	−178.39 (11)	C4A—C4B—C5—C6	177.60 (11)
C2—C1—C9A—C4A	0.87 (16)	C8A—C4B—C5—C6	−0.89 (16)
O1—C1—C9A—C4A	179.47 (11)	C4A—C4B—C8A—C8	−177.31 (10)
O1—C1—C9A—N9	0.21 (19)	C4B—C5—C6—O2	−179.27 (11)
C1—C2—C3—C4	54.63 (13)	C4B—C5—C6—C7	−0.24 (16)
C2—C3—C4—C4A	−49.23 (12)	O2—C6—C7—C8	179.92 (11)
C3—C4—C4A—C4B	−156.37 (11)	C5—C6—C7—C8	0.80 (18)
C3—C4—C4A—C9A	22.25 (14)	C6—C7—C8—C8A	−0.17 (17)
C4—C4A—C4B—C5	−0.7 (2)	C7—C8—C8A—N9	−178.45 (11)
C4B—C4A—C9A—C1	−178.62 (10)	C7—C8—C8A—C4B	−0.97 (16)
C4—C4A—C9A—C1	2.47 (17)		

Symmetry codes: (i) −*x*+1, −*y*+1, −*z*+1; (ii) −*x*+1, −*y*+1, −*z*+2; (iii) *x*−1, *y*, *z*−1; (iv) *x*+1, −*y*+3/2, *z*+1/2; (v) *x*, −*y*+3/2, *z*+1/2; (vi) −*x*, −*y*+1, −*z*+1; (vii) *x*, −*y*+3/2, *z*−1/2; (viii) *x*−1, −*y*+3/2, *z*−1/2; (ix) *x*, *y*, *z*−1; (x) *x*+1, *y*, *z*+1; (xi) *x*, *y*, *z*+1.

### Hydrogen-bond geometry (Å, °)

**Table:**

*D*—H···*A*	*D*—H	H···*A*	*D*···*A*	*D*—H···*A*
N9—H9···O1^i^	0.948 (17)	1.918 (17)	2.8313 (14)	161.2 (15)
C2—H2A···O2^x^	0.99	2.52	3.4962 (15)	169
C4—H4B···Cg^v^	0.99	2.57	3.492 (1)	156

Symmetry codes: (i) −*x*+1, −*y*+1, −*z*+1; (x) *x*+1, *y*, *z*+1; (v) *x*, −*y*+3/2, *z*+1/2.
